# Large‐scale dark diversity estimates: new perspectives with combined methods

**DOI:** 10.1002/ece3.2371

**Published:** 2016-08-04

**Authors:** Argo Ronk, Francesco de Bello, Pavel Fibich, Meelis Pärtel

**Affiliations:** ^1^ Institute of Ecology and Earth SciencesUniversity of TartuLai 40Tartu51005Estonia; ^2^ Department of BotanyFaculty of ScienceUniversity of South BohemiaBranišovská 31370 05České BudějoviceCzech Republic

**Keywords:** Biomod, composite dark diversity, consensus dark diversity, co‐occurrence, frequency, prevalence, species distribution modeling

## Abstract

Large‐scale biodiversity studies can be more informative if observed diversity in a study site is accompanied by dark diversity, the set of absent although ecologically suitable species. Dark diversity methodology is still being developed and a comparison of different approaches is needed. We used plant data at two different scales (European and seven large regions) and compared dark diversity estimates from two mathematical methods: species co‐occurrence (SCO) and species distribution modeling (SDM). We used plant distribution data from the Atlas Florae Europaeae (50 × 50 km grid cells) and seven different European regions (10 × 10 km grid cells). Dark diversity was estimated by SCO and SDM for both datasets. We examined the relationship between the dark diversity sizes (type II regression) and the overlap in species composition (overlap coefficient). We tested the overlap probability according to the hypergeometric distribution. We combined the estimates of the two methods to determine consensus dark diversity and composite dark diversity. We tested whether dark diversity and completeness of site diversity (log ratio of observed and dark diversity) are related to various natural and anthropogenic factors differently than simple observed diversity. Both methods provided similar dark diversity sizes and distribution patterns; dark diversity is greater in southern Europe. The regression line, however, deviated from a 1:1 relationship. The species composition overlap of two methods was about 75%, which is much greater than expected by chance. Both consensus and composite dark diversity estimates showed similar distribution patterns. Both dark diversity and completeness measures exhibit relationships to natural and anthropogenic factors different than those exhibited by observed richness. In summary, dark diversity revealed new biodiversity patterns which were not evident when only observed diversity was examined. A new perspective in dark diversity studies can incorporate a combination of methods.

## Introduction

Analysis of large‐scale species richness patterns is an important tool for ecology, biogeography and conservation biology (Gaston 2000). An important facet to the study of diversity patterns can be obtained by including the set of species that could potentially inhabit a study site but are currently absent – the dark diversity (Pärtel et al. 2011a; Ronk et al. 2015), that is, the absent portion of the site‐specific (i.e., both abiotic and biotic filtered) species pool (Cornell and Harrison 2014; Zobel 2016). Although suitable methods to estimate dark diversity are being developed, few attempts have been made to compare different methods (e.g., Dupré 2000; de Bello et al. 2016; Lewis et al. 2016b). Here we compare the estimates of dark diversity resulting from two different mathematical methods and how each relates to various natural and anthropogenic factors. We use vascular plant data at the continental scale, but our approaches can be applied to other organisms and at any spatial scale.

Dark diversity cannot be measured directly from local plant inventories (as opposed to observed species richness), rather it is estimated indirectly (Pärtel et al. 2011a). In particular, dark diversity can be detected from a broader view that incorporates data on the regional distribution and environmental preferences of species. For example, a species could be absent from a site (at large scale due to changes in environmental conditions or dispersal limitation or at small scale due to competition with other species or local stochastic events) but may still be present in the surrounding region (thereby constituting part of the species pool for that site) and potentially available to recolonize if suitable conditions recur. There can be also a concern whether species included in dark diversity are truly absent or have merely eluded detection (Pärtel et al. 2013). For example, when studying a 1 × 1 m vegetation plot once, species could have just been dormant. Similarly, published species’ distribution maps should be considered reliable although not perfect. Thus, dark diversity studies have same constraints as other biodiversity research and we should keep in mind the possibility that some species in estimated dark diversity have actually eluded sampling.

Dark diversity and the species pool concept have often been applied at the community (habitat type) scale (Lessard et al. 2012; de Bello et al. 2016; Lewis et al. 2016b), but dark diversity, as the complement of observed species richness, can be applied irrespective of scale (Pärtel et al. 2011a; Ronk et al. 2015). For example, we can study a 1 × 1 m plot in a grassland and determine which species are absent although ecologically suitable and present in the surrounding. Similarly, we can investigate a 1‐ha plot and look the landscape in the surrounding and determine which species in the surrounding area can potentially inhabit the plot. The plots in both examples inarguably contain a degree of environmental variability (only a point is completely homogeneous). At larger scales used in biogeography and macroecology (e.g., 10 × 10 km or 50 × 50 km), we deal with a combination of different traditionally distinguished habitat types and have a specific distribution of ecological conditions. A combination of traditional habitat types (e.g., boreal forest, raised bog, calcareous grassland) can still be considered as a habitat filter. Note that the term “species pool” was first used by MacArthur and Wilson (1967) in island biogeography theory in which it meant terrestrial habitat (in contrast to marine habitat), and the paper that introduced the dark diversity idea presented an example in which dark diversity was found a 100 × 100 km scale (Pärtel et al. 2011a). Even if a study site contains a mosaic of different ecological conditions, we can still find many other locations in the surrounding with similar habitat patterns and species compositions. We can summarize that a species from the surrounding region belongs to dark diversity if at least some part of the study site is ecologically suitable for that species. Thus, we acknowledge that dark diversity can be applied at smaller scales, but it is not limited to specific scales.

Scientists and nature conservationists can be more informed on the condition of biodiversity when we also explore dark diversity (Lewis et al. 2016a). From a nature conservation perspective, species “moving” from observed to dark diversity could indicate that local extinctions are occurring at the site. On the other hand, dark diversity can be seen as a buffer for protected areas. If species of interest are still in dark diversity, those species are still present in the region and can potentially disperse to protected areas (Lewis et al. 2016a). Dark diversity is especially informative when studied together with observed species richness. For example, we could have two sites which both have high species richness, but with low dark diversity in one site and high dark diversity in the other. At a relative scale, these sites will exhibit different realizations of their site‐specific species pool. This difference between sites would not have been evident from species richness alone. Completeness of site diversity index is defined as the log ratio between observed species richness and dark diversity and shows how much of the site‐specific species pool is actually realized at the site (Pärtel et al. 2013; Ronk et al. 2015). Dark diversity and completeness of site diversity can advance our understanding of how biodiversity is related to different natural and anthropogenic factors. For example, Ronk et al. (2015) found no relationship between completeness of site diversity and latitude because both observed richness and species pool exhibit a strong latitudinal gradient. By contrast, mountainous areas tended to be more complete than their surrounding areas suggesting lower human influence in mountainous areas at the European scale. It is unknown whether dark diversity and completeness estimates by different methods can provide unique information about diversity patterns when compared to observed species richness.

Dark diversity can include species that have not yet dispersed to the study site or that are temporarily absent due to stochastic changes in the study site (Pärtel et al. 2011a). In order to assign a species to dark diversity, the absent species must have a reasonable probability to disperse to the study site and be able to tolerate the local environmental conditions (at least somewhere within the study site). To determine which species can be assigned to dark diversity, we need to apply a series of dispersal and environmental filters (Ronk et al. 2015). Dispersal filters can be determined geographically. For example, some species from the high Alps may be able to tolerate environmental conditions near the Arctic. However, these environmentally similar regions are separated by other types of habitat, thereby rendering migration across these regions unlikely. Therefore, only species that occupy neighboring sites should be considered potential candidates for dark diversity (Ronk et al. 2015). Environmental filters can be based on different criteria, for example, using expert opinions, experiments, or mathematical algorithms (Pärtel et al. 2013). At small scales, all methods are possible; local experts can suggest which species can inhabit a site, compiled databases on habitat requirements (e.g., Ellenberg indicator values) can be used, or organisms can be introduced experimentally and their success observed. Mathematical approaches are more appropriate at large scales, because expertise is likely inadequate and the study extent and grain size is unsuitable for introduction experiments.

Each mathematical method has its own limitations and it is therefore prudent to maintain several methods in the dark diversity toolbox. The species co‐occurrence (SCO) approach has already been used to quantify environmentally suitable absent species at the European scale (Ronk et al. 2015). This method uses a large species × sites matrix to predict species’ ecological suitability based on the co‐occurrence of a given species with other species. Another promising approach to estimate dark diversity is species distribution modeling (SDM) (Pärtel et al. 2013). Species distribution models (SDM) are widely used in ecology and nature conservation (Elith et al. 2006; Thuiller et al. 2009). Methods of SDM have also developed greatly over the last decade (Thuiller 2003; Thuiller et al. 2009). Traditionally, SDM has been used to estimate the environmental niches of species and to project their potential distributional ranges using environmental variables and actual distribution data. Although SDM and SCO techniques serve a similar aim, to predict species occurrence at sites where species are previously absent although potentially suitable, they differ in methodology and the data type needed for modeling. Therefore, these methods could complement each other at large‐scale studies. Moreover, combining different methods could provide semiquantitative dark diversity estimates (species estimated by both or at least one method).

Whereas the SCO method requires only information on species presence and absences at sites (species × site data matrixes), SDM method also needs information on environmental conditions throughout the study region. This method uses sites where a species is present to find other sites which share similar environmental conditions but where the species is absent (Thuiller et al. 2009). Thus, the SCO method can be seen as a community approach (Ovaskainen and Soininen 2011), because the method uses a complete set of species co‐occurrences (De Caceres and Legendre 2008). Therefore, the SCO method could be well suited to testing hypotheses concerning community assembly and diversity maintenance; when applied at smaller scales, it might indicate biotic interactions (Riibak et al. 2015). By contrast, SDM can be seen as a species‐specific approach; an environmental envelope is developed for each species to predict its suitability at other sites. Both methods may be sensitive to species frequency characteristics in the dataset. Thus, a combination of these methods might complement each other.

Dark diversity has been usually considered a binary variable; species either belong to dark diversity or not (Pärtel et al. 2011a; Ronk et al. 2015). However, it would be beneficial to define dark diversity quantitatively using fuzzy set principles (Pärtel et al. 2011b), as in recently published biodiversity studies (Karger et al. 2016; Lessard et al. 2016). A step toward this would also be the combination of different dark diversity methods. This would allow for *consensus dark diversity* and *composite dark diversity*, defined as species which are assigned to dark diversity by both methods or by one method, respectively.

In this study, we explore two mathematical methods to estimate large‐scale dark diversity at two scales, the European‐wide and in more detail in seven regions within Europe. We aim to determine (1) whether SCO and SDM methods give similar dark diversity estimates; (2) the extent and significance of overlap between species composition in these dark diversities estimates; (3) whether consensus and composite dark diversity (one method or both methods) form different distribution patterns; and (4) whether different estimates of dark diversity and completeness of site diversity are related to observed richness, and whether they differ in their relations to various natural and anthropogenic factors.

## Material and methods

### Data

We used different a European‐wide and seven regional datasets for this study. For the European‐wide analyses, we used “Atlas Florae Europaeae” (Jalas and Suominen 1972; Jalas et al. 1996, 1999; Kurtto et al. 2004), in accordance with the official license purchased from the Finnish Museum of Natural History (http://www.luomus.fi/en/database-atlas-florae-europaeae). This atlas contains presence–absence distribution data for 4123 species or subspecies in Universal Transverse Mercator (UTM) grid cells with a resolution of 50 × 50 km. The study area was delimited in the east along the political boundaries of the Russian Federation, Ukraine, Belarus, and Moldova. This restriction was used because Atlas Florae Europaeae contains incomplete data in these regions (Manne and Williams 2003; Nogués‐Bravo and Araújo 2006). Remote islands (Azores, Canary, and Svalbard) were also excluded because most of these grid cells contain ocean. These delineations of the area reflect a trade‐off between geographical coverage and data quality. The European‐wide analyses contained 2486 grid cells.

For regional analyses, we used seven different areas from Europe with plant atlases freely available online: Finland (Lampinen and Lahti 2013, http://www.luomus.fi/kasviatlas/), Estonia (http://efloora.ut.ee/Eesti/index.html), Britain and Ireland (http://www.brc.ac.uk/plantatlas/), the Netherlands (http://soortenbank.nl/), Germany (http://www.floraweb.de/), Switzerland (http://www.infoflora.ch/de/flora/art-abfragen.html), and Catalonia (http://www.floracatalana.net/). Data were extracted from the web pages using the function “scan” in the R base package (R Core Team 2015). Most of these online atlases have a spatial resolution of ca 10 × 10 km; although the German atlas features a roughly 12 × 11 km grid cell size, the Dutch and Swiss atlases have a spatial resolution of 5 × 5 km. We aggregated the 5 × 5 km spatial resolution to 10 × 10 km to better match the scale of other regions. Altogether, the seven regions contained 12,606 grid cells. We considered each grid cell as one study site with defined observed richness, species composition, and environmental conditions.

Species distribution modeling requires environmental data at the same resolution as that of species presences and absences (Austin 2007). Large‐scale species distribution patterns often depend on climatic factors such as temperature and precipitation. Recent evidence has shown that climatic factors alone are often sufficient to predict species distributions at large scales (Bucklin et al. 2015). We used mean annual temperature, temperature seasonality (annual range), mean annual precipitation, and precipitation seasonality (annual range). Climatic data were averaged to each 50 × 50 and 10 × 10 km grid cell from 5′ data derived from WorldClim (http://www.worldclim.org/; Hijmans et al. 2005). European landscapes, however, have been strongly modified by human land use; therefore, the dominant land cover type based on CORINE 2006 level 2 (http://www.eea.europa.eu/data-and-maps/data/corine-land-cover-2006-raster; Bossard et al. 2000) was included. A lack of CORINE land cover data resulted in some regions (e.g., Greece) being excluded from the analyses.

### Estimating dark diversity

We merged subspecies at the species level and resolved taxonomic synonyms according to the Plant List database (http://www.theplantlist.org/). For both European and regional scales, we used only species that occurred in at least in twenty grid cells (each region was treated separately). Environmental suitability of species for dark diversity was estimated by SCO and SDM methods.

Dark diversity estimation (count of number of species) by SCO is based on an index that quantifies the probability of joint occurrence of a particular species with other species (Beals 1984; Ewald 2002; Ronk et al. 2015; Lewis et al. 2016b):

$$P_{ij}=\left(1/S_{i}\right)\sum_{k}N_{jk}I_{ik}/N_{k}$$

where *P*_*ij*_ is the probability that species *j* occurs in site *i*,* S*_*i*_ is the number of species at site *i* (excluding species *j*), *N*_*jk*_ is the number of joint occurrences of species *j* and *k* (*j* ≠ *k*), *I*_*ik*_ is the incidence (0 or 1) of species *k* in site *i*, and *N*_*k*_ is the number of occurrences of species *k*. Each species was assigned a threshold value for inclusion into dark diversity. A species was included in dark diversity when it was absent from a grid cell and its occurrence probability was greater than 5% of the values in grid cells where the species was actually present. Observed species richness and species composition (species that were actually recorded in the grid cell) were treated as presence/absence; we did not calculate occurrence probabilities for observed species.

Species distribution modeling can be performed by different algorithms. Some algorithms perform generally better in specific circumstances, but there is no superior method (Segurado and Araújo 2004; Pearson et al. 2006; Austin 2007). We used generalized additive models, which have often performed well (Elith et al. 2006; Meynard and Quinn 2007), especially with large sample sizes (Thibaud et al. 2014). Generalized additive models were fitted using logit as the link function and a binomial error distribution. As commonly practiced, we used data splitting (80% of the data was used for calibration and 20% for evaluation), which we ran on different random selections. Resultant species occurrence probabilities were transformed into species presences and absences using a threshold giving the best quality of predictions, evaluated as the best score of true skill statistic (TSS, Allouche et al. 2006). Therefore, depending on the method (SCO or SDM), we used different thresholds for inclusion to dark diversity. We did not use an ensemble forecast of different methods as different methods have their own limitations and are unable to work with all species. As a consequence, such a technique omits many species from the analyses.

In order to account for species dispersal probability and biogeographical history, we applied two additional spatial filters along with the environmental filters (SCO or SDM). For dispersal probability, we set a geographical filter that excluded from dark diversity species not found within a 500‐km radius at the European scale (50 × 50 km) and a 300‐km radius at the regional scale (10 × 10 km). This makes spatial and temporal scales comparable as species within 300 km can potentially arrive in a shorter time span than species within 500 km. Sensitivity analyses, however, confirm that these two radii produce strongly correlated dark diversity estimates (Ronk et al. 2015). To account for common biogeographical history, we excluded from dark diversity all species not found within the biogeographical region, as defined by the dispersion field technique (Graves and Rahbek 2005). For this technique, we used those grid cells containing >50% of the species from the study grid cell (Carstensen et al. 2013). In other words, although species could be assigned dark diversity based on environmental criteria, species must also be present within a set radius and must occur sites that share similar biogeographical history as the study site (Ronk et al. 2015).

Completeness of site diversity was calculated *sensu* community completeness index according to Pärtel et al. (2013) as ln(observed richness/dark diversity).

In order to obtain semiquantitative values of dark diversity, we counted species which were assigned to dark diversity by both methods (*consensus dark diversity*), or by one method (*composite dark diversity*). Dark diversity calculations were performed using R ver. 3.0.2 (R Development Core Team). We used “BIOMOD2” (Thuiller et al. 2009) package (R package version 3.1‐25) for SDM and “vegan” (Oksanen et al. 2013) package (R package version 2.0‐10) for SCO.

### Comparisons of methods

We compared the results of the two methods using European‐wide and regional datasets (all regions combined). Dark diversity estimates by the SCO and SDM method were ln‐transformed and used as variables in type II regression calculated by the “lmodel2” function within (R package version 1.7‐2) “lmodel2” (Legendre 2014). This regression model does not assign independent and dependent variables and can be used if both variables are estimated. We extracted *R* ^2^ values and tested whether the major axis deviates from the 1:1 line (i.e., if the confidence intervals for the type II regression slopes include 1). The overlap coefficient was calculated to compare species composition of both methods (Kowalski 2011). This index is defined as the number of species common to both methods divided by the smaller dark diversity size of the two methods. Compared with many other indexes (e.g., Jaccard index), this index is not dependent on the size variation of the dark diversity estimates. Separate results for each region are given in Supplementary material (Appendix S1, Table S1). In order to test whether the overlap of two methods is different from random expectation, we calculated probability values for the observed and greater overlaps according to the hypergeometric distribution (Forbes et al. 2011). This discrete distribution describes the exact probability of overlap between two methods if we draw randomly without replacement *x*_1_ species (dark diversity size according to the first method) from a set of *N* species (number of species absent from the site), divided into two groups: species belonging to dark diversity according to the second method (*x*_2_), and the remainder (*N*‐*x*_2_ species). We used R function “phyper” (R Core Team 2015) and calculated mean and median *P*‐values over all sites and percentage of sites when *P* value was >0.05.

To test how dark diversity and completeness of site diversity estimates are related to observed species richness, we plotted different estimates of dark diversity (Supplementary material Appendix S2) and completeness of site diversities against observed species richness and calculated Pearson correlation coefficients (Appendix S3). In order to test the effects of various explanatory variables (climate, geological and soil properties, plant growth conditions, land‐use practices) on the diversity metrics, we first constructed principal components with “PCA” function within the package “FactoMineR” (R package version 1.29) (Husson et al. 2015). To correct for a possible bias in species richness due to reduced land surface in coastal cells, we used only grid cells that encompassed 100% land. For PCA analyses at the European scale, we were able to use 1214 grid cells and for regional scale 10,284 grid cells in which all explanatory variables had a numerical value. For European scale analyses, we used 31 different factors and for regional scale 22 different factors for which we had sufficient data and spatial resolution according to scale. For most variables, we used both mean and range. We constructed principal components for European (50 × 50 km) and regional (10 × 10 km) scales. For European scale, we chose the first three principal components, which altogether explained 54% of the variance. The first principal component had its strongest relationships with mean of temperature range, mean of altitudinal range, and mean of precipitation range. Therefore, we named PCA1 “heterogeneity”. The second principal component had its strongest relationships with mean soil organic matter, latitude, and mean pH. Therefore, we named PCA2 “latitude”. The third principal component had its strongest relationships with temperature and precipitation seasonality. Therefore, we named PCA3 “seasonality”. For regional scale, we chose the first two principal components, which altogether explained 51% of variance. The first principal component had its strongest relationships with latitude and mean of precipitation range. Therefore, we named PCA1 “latitude”. The second principal component had its strongest relationships with average altitude, altitudinal range, and annual temperature range. Therefore, we named PCA2 “heterogeneity”. Selected principal components were used as explanatory variables in linear mixed effect models constructed with the “lme” function within R package “nlme” (Pinheiro et al. 2015). All parameters were first standardized (mean 0, standard deviation 1). In regional analyses, we used region as a random factor in the models. We measured the effect of spatial autocorrelation in our models with Moran's *I* on normalized residuals of the simple models. Due to spatial autocorrelation, we fitted linear mixed effect models with exponential spatial correlation structure and a nugget value allowed (Dormann et al. 2007). We used z‐test to test differences between models estimates when the dependent variable was observed species richness or an estimate of dark diversity or a completeness of site diversity. Detailed information on explanatory variables and their contribution to principal components is available in Supplementary material (Appendices S4 and S5).

Our calculations of dark diversity are limited to the available data. Theoretically dark diversity at sites at the study area borders might be underestimated as the area for geographical species pool extends beyond the data limit. We estimated this effect for the European dataset. For this, we compared dark diversity sizes along sites next to the border (first site situated completely within the study area, where we would expect a boundary effect to be strongest) and sites 500 km from the eastern border (paired *t*‐test at *P* < 0.05). We found statistically significant boundary effect only for SDM method (*t* = −3.0, *P* = 0.006), although the average difference in dark diversity sizes between the compared sites was small (6%). SCO and combined dark diversity methods were not affected by a boundary effect.

## Results

**Figure 1:**
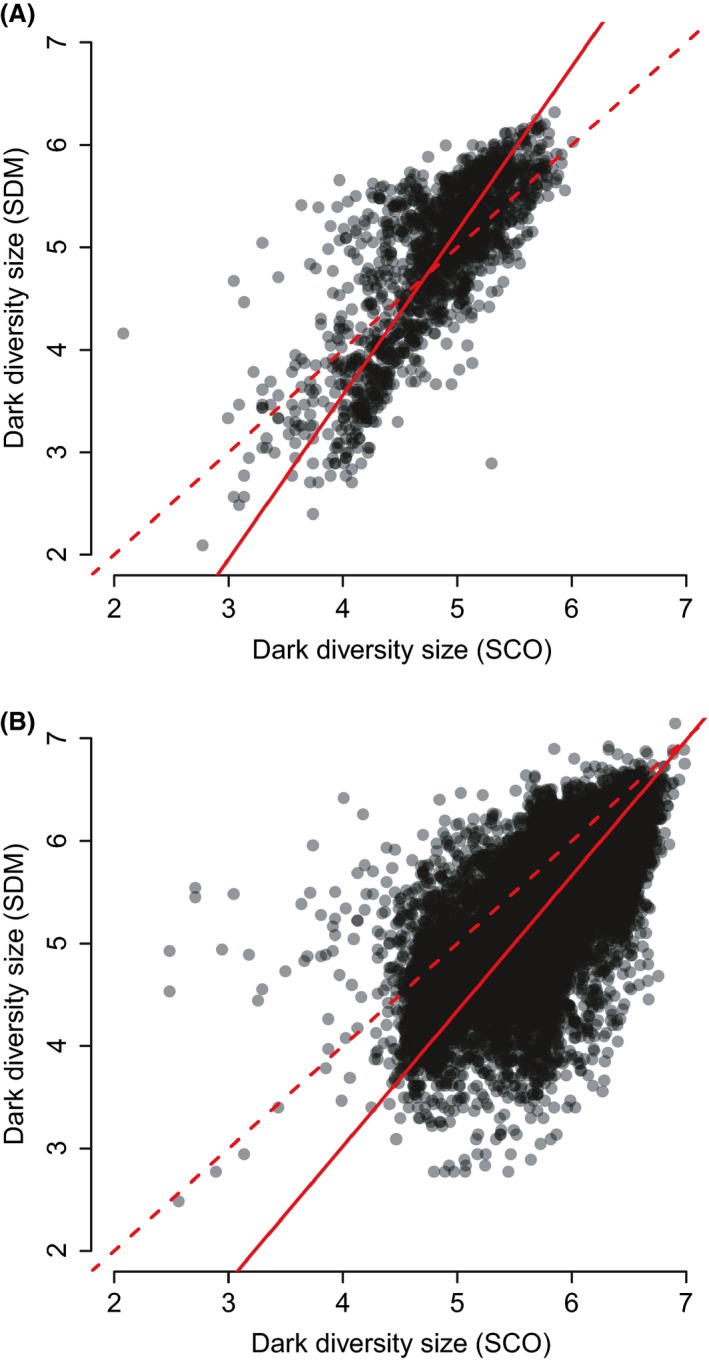
Relationship between dark diversity (ln‐transformed) by species co‐occurrence (SCO) and species distribution modeling (SDM) methods at (A) European scale (*R* ^2^ = 0.61, slope = 1.6, 95% CI 1.5 and 1.7, *P* < 0.001) and (B) regional scale *R* ^2^ = 0.48, slope = 1.32, 95% CI 1.29 and 1.35, *P* < 0.001). Solid line represents type II regression line and dashed line 1:1 relationship. Regression lines deviate from 1:1 lines.

Estimates of dark diversity exhibit strong positive correlation at both scales, but type II regression slopes differed from the 1:1 line (Fig. 1). On average, SCO and SDM assigned similar sizes of dark diversity at the European scale, but at the regional scale SCO assigned more dark diversity than SDM. As a result, at the European scale both methods were fairly similar at intermediate dark diversity values; at the regional scale, the difference between dark diversity estimates was greater at low values but roughly agreed at larger values. Dark diversity values were positively correlated with observed species richness (*r* = 0.27 …0.42 at the European scale, 0.50 … 0.70 at the regional scale, Appendix S2). Completeness of site diversity was positively correlated with observed richness (*r* = 0.27 … 0.47 at the European scale, 0.38…0.61 at the regional scale, Appendix S3). Species composition overlap of two methods was about 75% at both European and regional scales (the first and the third quartiles at the European scale are 63% and 78% and at the regional scale 64% and 84%). We found that the overlap between two methods was greater than random expectation at both European scale (average and median *P*‐values <0.001; percentage when *P* value was >0.05 over all sites was 0.1%) and regional scale (average and median *P*‐values <0.001; percentage when *P* value was >0.05 over all sites was 2.9%).

**Figure 2:**
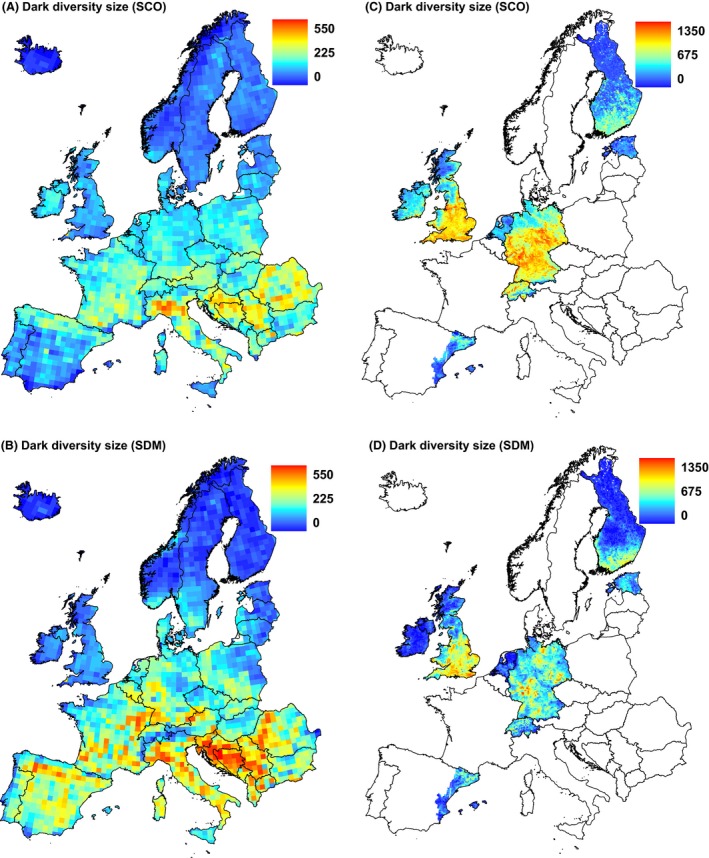
Dark diversity estimates at the European scale calculated with (A) species co‐occurrence (SCO) and (B) species distribution modeling (SDM) method; and at the regional scale (C) species co‐occurrence and (D) species distribution modeling. For comparison, we used the same scale for both dark diversity methods in A and B, and C and D, respectively. Projection: Lambert azimuthal equal area.

**Figure 3:**
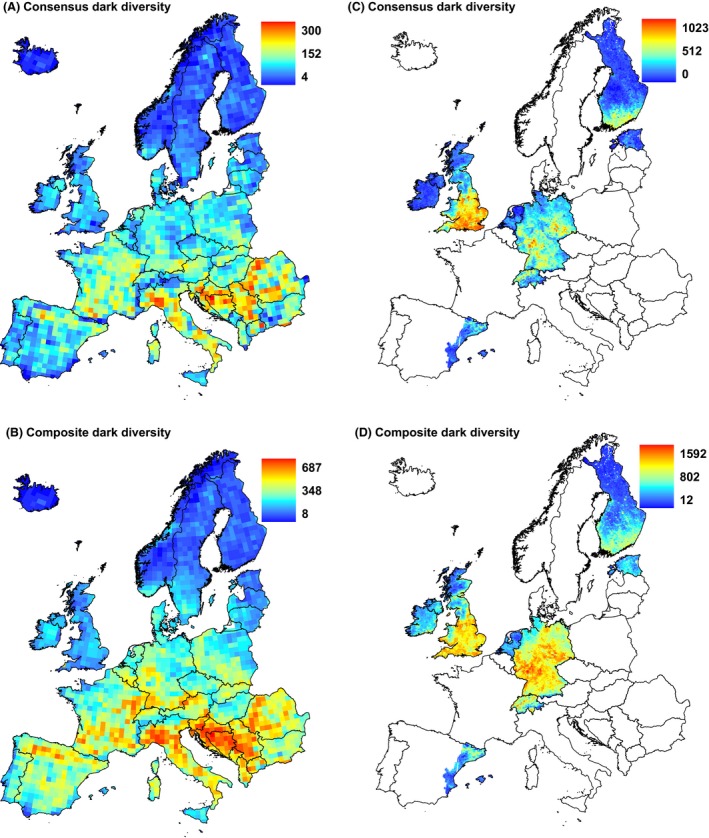
Consensus and composite dark diversity estimates (species predicted by both or one method) at the European scale (A) consensus dark diversity, (B) composite dark diversity, and at the regional scale (C) consensus dark diversity and (D) composite dark diversity. Note that each map has own scale. Projection: Lambert azimuthal equal area.

At the European scale, SCO and SDM methods both showed that dark diversity is low in north Europe and high in south Europe (Fig. 2), although in some areas, for example, in the Iberian Peninsula, SCO assigned less dark diversity than SDM. Consensus and composite dark diversity showed relatively similar patterns (Fig. 3). Consensus dark diversity by both methods estimated dark diversity to be greatest in southern Europe. Compared with consensus dark diversity, the composite dark diversity showed rather greater dark diversity in central Europe and in the Iberian Peninsula. Heterogeneity (PCA1) showed differences between observed species richness and dark diversity estimates: All dark diversity estimates were significantly weaker than for observed species richness. Dark diversity calculated by SDM method and composite dark diversity both showed positive relationships with latitude (PCA2), and these relationships differed significantly from that of observed species richness (no relationship in spatially informed model). Seasonality (PCA3) showed the greatest differences between observed species richness and dark diversity estimates: Observed species richness was related positively whereas dark diversity estimates related negatively (Table 1). The regional scale shows also a similar pattern with some differences, for example, dark diversity by SCO is less than by SDM in north Germany. Dark diversity estimates showed similar negative relationships with latitude (PCA1) and heterogeneity (PCA2, Table 2), and all dark diversity estimates had significantly different relationships than patterns from observed species richness.

**Table 1:** Coefficients of linear mixed effect model relating observed species richness, different estimates of dark diversity and completeness of site diversity to three principal components derived from various natural and anthropogenic factors at the European scale. Standard error is given in parentheses. Difference between coefficients from observed richness and from an estimate of dark diversity or completeness was evaluated by *z*‐test, and significantly different results are in bold. Full model results are given in Supporting Information (Appendix S6)

	PCA1 (heterogeneity)	PCA2 (latitude)	PCA3 (seasonality)
Observed richness	0.139 (0.014)	−0.033 (0.022)	0.084 (0.028)
Dark diversity (SCO)	**−0.001 (0.014)**	0.019 (0.023)	**−0.145 (0.030)**
Dark diversity (SDM)	**0.022 (0.014)**	**0.100 (0.024)**	**−0.097 (0.032)**
Dark diversity (Consensus)	**−0.009 (0.016)**	−0.050 (0.026)	**−0.165 (0.035)**
Dark diversity (Composite)	**0.024 (0.012)**	**0.113 (0.021)**	**−0.111 (0.028)**
Completeness (SCO)	**0.082 (0.017)**	−0.004 (0.025)	0.151 (0.034)
Completeness (SDM)	**0.049 (0.016)**	−0.054 (0.026)	0.127 (0.035)
Completeness (Consensus)	**0.074 (0.016)**	0.050 (0.023)	0.130 (0.032)
Completeness (Composite)	**0.060 (0.016)**	**−0.062 (0.026)**	0.141 (0.034)

**Table 2:** Coefficients of linear mixed effect model relating observed species richness, different estimates of dark diversity and completeness of site diversity to principal components from various natural and anthropogenic factors at the regional scale. Standard error is given in parentheses. Difference between coefficients from the observed richness and from an estimate of dark diversity or completeness was evaluated by *z*‐test, and significantly different results are in bold. Full model results are given in Supporting Information (Appendix S6)

	PCA1 (latitude)	PCA2 (heterogeneity)
Observed richness	−0.120 (0.015)	−0.035 (0.011)
Dark diversity (SCO)	−0.118 (0.011)	**−0.150 (0.008)**
Dark diversity (SDM)	**0.004 (0.011)**	**−0.100 (0.008)**
Dark diversity (Consensus)	**−0.038 (0.011)**	**−0.119 (0.008)**
Dark diversity (Composite)	**−0.072 (0.009)**	**−0.133 (0.006)**
Completeness (SCO)	**−0.038 (0.021)**	**0.100 (0.015)**
Completeness (SDM)	−0.110 (0.021)	**0.056 (0.015)**
Completeness (Consensus)	−0.075 (0.020)	**0.082 (0.015)**
Completeness (Composite)	−0.08 (0.022)	**0.075 (0.016)**

**Figure 4:**
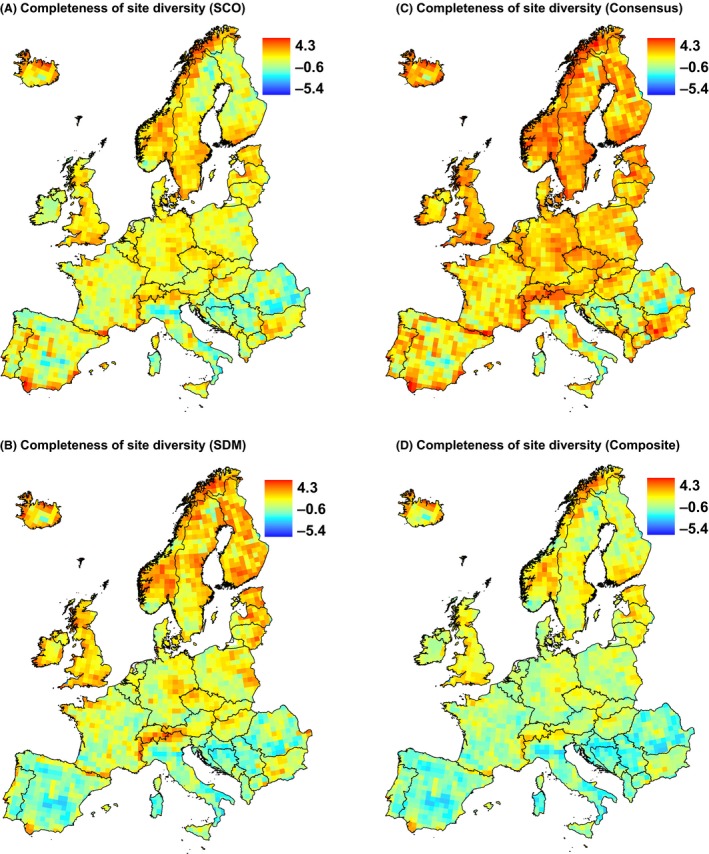
Completeness of site diversity at the European scale calculated with (A) species co‐occurrence and (B) species distribution modeling method, and (C) consensus of used methods and (D) composite of used methods. For comparison, we used the same scale for all maps. Projection: Lambert azimuthal equal area.

**Figure 5:**
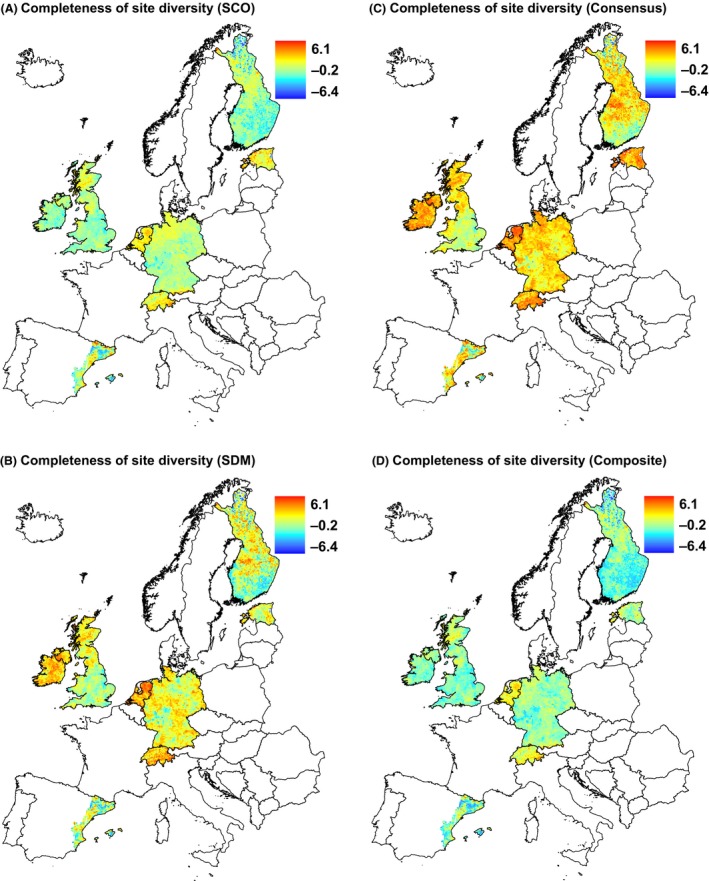
Completeness of site diversity at the regional scale calculated with (A) species co‐occurrence and (B) species distribution modeling method, and (C) consensus of used methods and (D) composite of used methods. For comparison, we used the same scale for all maps. Projection: Lambert azimuthal equal area.

Both SCO and SDM methods resulted in similar scattered patterns of completeness of site diversity across Europe (Fig. 4). Relatively complete sites can be found in both in north and south Europe. Estimates of completeness of site diversities increased with increasing heterogeneity (PCA1); the trend, however, was significantly weaker compared with the positive relationship between heterogeneity and observed species richness (Table 1). In general, completeness estimates were not significantly different from observed species richness results, which showed no strong relationship with latitude (PCA2). There were positive relationships with seasonality (PCA3), similar to the relationship with species richness (Table 1). At the regional scale, SCO and SDM methods show in general concordant patterns with some differences, for example, sites appear more complete by SDM than by SCO in some regions in Germany and Ireland (Fig. 5). Different estimates of completeness of site diversities decreased with increasing latitude (PCA1), but the relationships were generally not stronger for observed species richness (except for SCO completeness, Table 2). All estimates of completeness of site diversities showed positive relationship with heterogeneity (PCA2), which was significantly different than that of observed species richness (negative relationship).

## Discussion

Dark diversity (ecologically suitable absent species in a study site) can complement commonly used observed species richness in studies on biodiversity and nature conservation (Pärtel et al. 2011a; Ronk et al. 2015). Although dark diversity can be estimated by different means (Pärtel et al. 2013), the methodical toolbox is still being developed and properties of mathematical methods usable at large spatial scales need exploration. In biodiversity studies, ensemble forecasts from different models have shown promise, because different techniques can potentially carry unique information (Araújo and New 2007). A similar philosophy can be applied to dark diversity estimates as well. We used plant data at two different biogeographical scales (European‐wide and seven regions) and measured dark diversity by two mathematical methods: SCO and SDM. Estimates of dark diversity sizes exhibited strong positive correlation at both scales despite deviating from the 1:1 line. Clearly, each method carried unique information. The overlap coefficient of two methods was 75%. By combining these two methods, we can attain consensus dark diversity estimates (species predicted by both methods) and composite dark diversity estimates (species predicted by one method), each of which can serve different purposes. Dark diversity and completeness of site diversity estimates added new information to diversity patterns when related to various natural and anthropogenic factors.

When relating dark diversity estimates of both methods, the type II regression line deviated from a 1:1 relationship, showing that the two methods are not totally equivalent. At the European scale, SCO and SDM generally produced similar dark diversity estimates, especially at intermediate dark diversity sizes. At the regional scale, dark diversity was less according to SDM than SCO with closer agreement at greater dark diversity values. This difference could arise from the finer scale of the study systems, that is, factors other than climate might dictate species distribution used in SDM (Austin 2007). Also SCO likely captures within‐grid floristic variation better and therefore predicts greater richness for heterogeneous conditions with mixed floristic elements. Both SCO and SDM methods clearly agreed that dark diversity was larger in southern Europe and smaller in northern Europe, although with slight regional differences. A similar latitudinal pattern of dark diversity was found using the SCO method (Ronk et al. 2015). This suggests that either method can be used when the size of dark diversity is needed but not its composition. In addition, SDM requires checking for a possible boundary effect.

Species co‐occurrence and SDM methods gave an overlap coefficient of approximately 75%. This highly significant agreement on species composition was similar at both European and regional scale. The remaining 25% could arise from varying species frequency. SCO method uses only species presences and absences and needs “enough” co‐occurrences with other species to include a species in dark diversity. The SDM method has been found to perform rather poorly for common species with wide environmental niche (Elith et al. 2006).

Our results on the size and composition of dark diversity estimates by SCO and SDM indicates that both methods carry some unique information and can potentially complement each other. Dark diversity can be regarded as a fuzzy set in which species are granted probabilistic membership, although so far membership in dark diversity has been mostly applied as a binary variable (Pärtel et al. 2011b). Combinations of different methods enabled us to define dark diversity semiquantitatively by including species from both or one method: consensus and composite dark diversity estimates. Both estimates showed similar spatial patterns, although the distribution of composite dark diversity was smoother. As consensus dark diversity has greater confidence by having support from two methods, it could be preferred for nature conservation to decide whether a particular species should be included in the dark diversity of a site, for example, for restoration. By contrast, we suggest that composite dark diversity could be used for analyses of large‐scale diversity patterns as it contains more information from different methods.

Compared with observed richness, dark diversity and completeness of site diversity estimates added new information. Both were correlated with observe richness, but the correlation coefficients were not particularly great. While dark diversity estimates showed patchy geographical distributions at both the European scale and within regions, similar to observed richness (Ronk et al. 2015), the completeness of site diversity showed scattered patterns. It is crucial to know which natural and anthropogenic factors determine different aspects of diversity (Gaston 2000). We used a large number of explanatory parameters and were able to describe >50% of variation when reduced to a few principal components. Compared with observed richness, dark diversity or completeness estimates were in general similarly related to principal components, but with some significant differences. Consequently, processes that define how many species are actually found (observed richness) do not necessarily define how many species from the regional species pool are absent (dark diversity) or the proportion of present and absent species (completeness). Moreover, these measures depend on the spatial scale. For example, at the European scale (50 × 50 km), heterogeneity induces greater observed richness than completeness; the opposite is true at the regional scale (10 × 10 km).

In summary, the dark diversity toolbox is still being developed and examination is needed of the properties of different methods. Mathematical methods, in particular SCO and SDM, can be considered at large spatial scales. We found that both methods give comparable results but also carried unique information that complements observed richness. We suggest that the new perspective in dark diversity studies entails a combination of methods; this allows semiquantitative definition of dark diversity for different purposes.

## Conflict of Interest

None declared.

## Data Accessibility

Atlas Florae Europaeae database was used according to the official license purchased from the Finnish Museum of Natural History. All other databases are freely available in the Internet.

## Supporting information


**Appendix S1.** Separate analyses from different regions.
**Table S1.** Summary of dark diversity size estimates (ln transformed) from species co‐occurrence (SCO) and species distribution modelling (SDM) methods, relationship between dark diversity estimates (Type II regression slope having SCO on *x* axis and SDM on y axis with 95 CI in parentheses, and *R* ^2^), and species composition matching (Overlap coefficient); 25th and 75th quantiles are given in parentheses.
**Appendix S2.** Correlation between observed species richness and different dark diversity estimates.
**Figure S1.** Scatterplots of observed species richness and: A) dark diversity estimated only by species co‐occurrence (SCO) method (*t* = 15.0, df = 1590, *P* < 0.001, *r* = 0.35), B) dark diversity estimated only by species distribution modelling (SDM) method (*t* = 17.1, df = 1590, *P* < 0.001, *r* = 0.39), C) dark diversity (common species) estimated by both methods (*t* = 11.0, df = 1590, *P* < 0.001, *r* = 0.27), D) dark diversity (all species) estimated by both methods (*t* = 18.6, df = 1590, *P* < 0.001, *r* = 0.42), at the European scale (A,B,C,D) and E) dark diversity estimated only by species co‐occurrence (SCO) method (*t* = 94.0, df = 9313, *P* < 0.001, *r* = 0.7), F) dark diversity estimated only by species distribution modelling (SDM) method (*t* = 55.0, df = 9313, *P* < 0.001, *r* = 0.5), G) dark diversity (common species) estimated by both methods (*t* = 65.3, df = 9313, *P* < 0.001, *r* = 0.56), H) dark diversity (all species) estimated by both methods (*t* = 87.8, df = 9313, *P* < 0.001, *r* = 0.67) at the regional scale (E,F,G,H).
**Appendix S3.** Correlation between observed species richness and different completeness of site diversities.
**Figure S2.** Scatterplots of observed species richness and: A) completeness of site diversity estimated only by species co‐occurrence (SCO) method (*t* = 21.2, df = 1590, *P* < 0.001, *r* = 0.47), B) completeness of site diversity estimated only by species distribution modelling (SDM) method (*t* = 8.4, df = 1590, *P* < 0.001, *r* = 0.21), C) completeness of site diversity (common species) estimated by both methods (*t* = 18.3, df = 1590, *P* < 0.001, *r* = 0.42), D) completeness of site diversity (all species) estimated by both methods (*t* = 13.7, df = 1590, *P* < 0.001, *r* = 0.32), at the European scale (A,B,C,D) and E) completeness of site diversity estimated only by species co‐occurrence (SCO) method (*t* = 61.9, df = 9313, *P* < 0.001, *r* = 0.54), F) completeness of site diversity estimated only by species distribution modelling (SDM) method (*t* = 50.3, df = 9313, *P* < 0.001, *r* = 0.46), G) completeness of site diversity (common species) estimated by both methods (*t* = 39.8, df = 9313, *P* < 0.001, *r* = 0.38), H) completeness of site diversity (all species) estimated by both methods (*t* = 74.4, df = 9313, *P* < 0.001, *r* = 0.61) at the regional scale (E,F,G,H).
**Appendix S4.** Source and characteristics of the explanatory variables considered in this study.
**Table S2.** List of explanatory variables used in linear mixed effect models
**Appendix S5.** Correlation matrices from PCA analyses.
**Table S3.** Relationships between explanatory variables and first three principal components at the European scale.
**Table S4.** Relationships between explanatory variables and first two principal components at the regional scale.
**Appendix S6.** Spatially informed linear mixed effect model results — Observed species richness and different dark diversity and completeness of site diversity estimates (European and regional scale) related to natural and anthropogenic factors.
**Table S5.** Summary results of the spatially‐informed linear mixed effect model linking observed species richness to natural and anthropogenic factors at the European scale (df = 1213).
**Table S6.** Summary results of the spatially‐informed linear mixed effect model linking observed species richness to natural and anthropogenic factors at the regional scale (df = 10274).
**Table S7.** Summary results of the spatially‐informed linear mixed effect model linking dark diversity (SCO) to natural and anthropogenic factors at the European scale (df = 1213).
**Table S8.** Summary results of the spatially‐informed linear mixed effect model linking dark diversity (SCO) to natural and anthropogenic factors at the regional scale (df = 10274).
**Table S9.** Summary results of the spatially‐informed linear mixed effect model linking dark diversity (SDM) to natural and anthropogenic factors at the European scale (df = 1213).
**Table S10.** Summary results of the spatially‐informed linear mixed effect model linking dark diversity (SDM) to natural and anthropogenic factors at the regional scale (df = 10274).
**Table S11.** Summary results of the spatially‐informed linear mixed effect model linking consensus dark diversity to natural and anthropogenic factors at the European scale (df = 1213).
**Table S12.** Summary results of the spatially‐informed linear mixed effect model linking consensus dark diversity to natural and anthropogenic factors at the regional scale (df = 10274).
**Table S13.** Summary results of the spatially‐informed linear mixed effect model linking composite dark diversity to natural and anthropogenic factors at the European scale (df = 1213).
**Table S14.** Summary results of the spatially‐informed linear mixed effect model linking composite dark diversity to natural and anthropogenic factors at the regional scale (df = 10274).
**Table S15.** Summary results of the spatially‐informed linear mixed effect model linking completeness of site diversity (SCO) to natural and anthropogenic factors at the European scale (df = 1213).
**Table S16.** Summary results of the spatially‐informed linear mixed effect model linking completeness of site diversity (SCO) to natural and anthropogenic factors at the regional scale (df = 10274).
**Table S17.** Summary results of the spatially‐informed linear mixed effect model linking completeness of site diversity (SDM) to natural and anthropogenic factors at the European scale (df = 1213).
**Table S18.** Summary results of the spatially‐informed linear mixed effect model linking completeness of site diversity (SDM) to natural and anthropogenic factors at the regional scale (df = 10274).
**Table S19.** Summary results of the spatially‐informed linear mixed effect model linking consensus completeness of site diversity to natural and anthropogenic factors at the European scale (df = 1213).
**Table S20.** Summary results of the spatially‐informed linear mixed effect model linking consensus completeness of site diversity to natural and anthropogenic factors at the regional scale (df = 10274).
**Table S21.** Summary results of the spatially‐informed linear mixed effect model linking composite completeness of site diversity to natural and anthropogenic factors at the European scale (df = 1213).
**Table S22.** Summary results of the spatially‐informed linear mixed effect model linking composite completeness of site diversity to natural and anthropogenic factors at the regional scale (df = 10274).
