# Supplementary material for: Large‐scale dark diversity estimates: new perspectives with combined methods
Source: Ecol Evol. 2016 Aug 4;6(17):6266–81. doi: 10.1002/ece3.2371 (PMC5016647; doi:10.1002/ece3.2371)
Supplement: Supplementary file 1 — Appendix S1. Separate analyses from different regions. Table S1. Summary of dark diversity size estimates (ln transformed) from species co‐occurrence (SCO) and species distribution modelling (SDM) methods, relationship between dark diversity estimates (Type II regression slope having SCO on x axis and SDM on y axis with 95 CI in parentheses, and R 2), and species composition matching (Overlap coefficient); 25th and 75th quantiles are given in parentheses. Appendix S2. Correlation between observed species richness and different dark diversity estimates. Figure S1. Scatterplots of observed species richness and: A) dark diversity estimated only by species co‐occurrence (SCO) method (t = 15.0, df = 1590, P < 0.001, r = 0.35), B) dark diversity estimated only by species distribution modelling (SDM) method (t = 17.1, df = 1590, P < 0.001, r = 0.39), C) dark diversity (common species) estimated by both methods (t = 11.0, df = 1590, P < 0.001, r = 0.27), D) dark diversity (all species) estimated by both methods (t = 18.6, df = 1590, P < 0.001, r = 0.42), at the European scale (A,B,C,D) and E) dark diversity estimated only by species co‐occurrence (SCO) method (t = 94.0, df = 9313, P < 0.001, r = 0.7), F) dark diversity estimated only by species distribution modelling (SDM) method (t = 55.0, df = 9313, P < 0.001, r = 0.5), G) dark diversity (common species) estimated by both methods (t = 65.3, df = 9313, P < 0.001, r = 0.56), H) dark diversity (all species) estimated by both methods (t = 87.8, df = 9313, P < 0.001, r = 0.67) at the regional scale (E,F,G,H). Appendix S3. Correlation between observed species richness and different completeness of site diversities. Figure S2. Scatterplots of observed species richness and: A) completeness of site diversity estimated only by species co‐occurrence (SCO) method (t = 21.2, df = 1590, P < 0.001, r = 0.47), B) completeness of site diversity estimated only by species distribution modelling (SDM) method (t = 8.4, df = 1590, P < 0.001, r [file ECE3-6-6266-s001.pdf]

Supplementary material

Appendix S1. Separate analyses from different regions.

Table S1 Summary of dark diversity size estimates (ln transformed) from species co-occurrence (SCO) and species distribution modelling (SDM) methods, relationship between dark diversity estimates (Type II regression slope having SCO on x axis and SDM on y axis with 95 CI in parentheses, and R2), and species composition matching (Overlap coefficient); 25th and 75th quantiles are given in parentheses.

	Count of grid cells	Mean dark diversity by SCO	Mean dark diversity by SDM	R2	Slope	Species composition matching
Finland	2744	5.2 (4.8; 5.7)	4.9 (4.3; 5.4)	0.37	1.7 (1.6;1.8)	0.73 (0.61;0.83)
Estonia	379	5.1 (4.9; 5.4)	5.4 (5.1; 5.7)	0.10	0.6 (0.4;0.8)	0.45 (0.36;0.57)
British Isles	2459	6.0 (5.6; 6.4)	5.4 (4.7; 6.3)	0.61	2.0 (1.9;2.1)	0.80 (0.71;0.86)
Netherlands	329	5.4 (5.2; 5.6)	4.6 (4.1; 5.1)	0.16	4.9 (3.9;6.5)	0.80 (0.69;0.89)
Germany	2810	6.3 (6.1; 6.5)	5.8 (5.6, 6.1)	0.09	2.1 (1.9;2.4)	0.74 (0.64; 0.82)
Switzerland	482	6.1 (5.9; 6.4)	5.6 (5.2; 6.0)	0.20	1.2 (1.0;1.4)	0.82 (0.71; 0.9)
Catalonia	635	5.2 (4.9; 5.7)	5.2 (4.8; 5.7)	0.35	1.2 (1.1;1.3)	0.67 (0.61;0.73)

Appendix S2. Correlation between observed species richness and different dark diversity estimates.

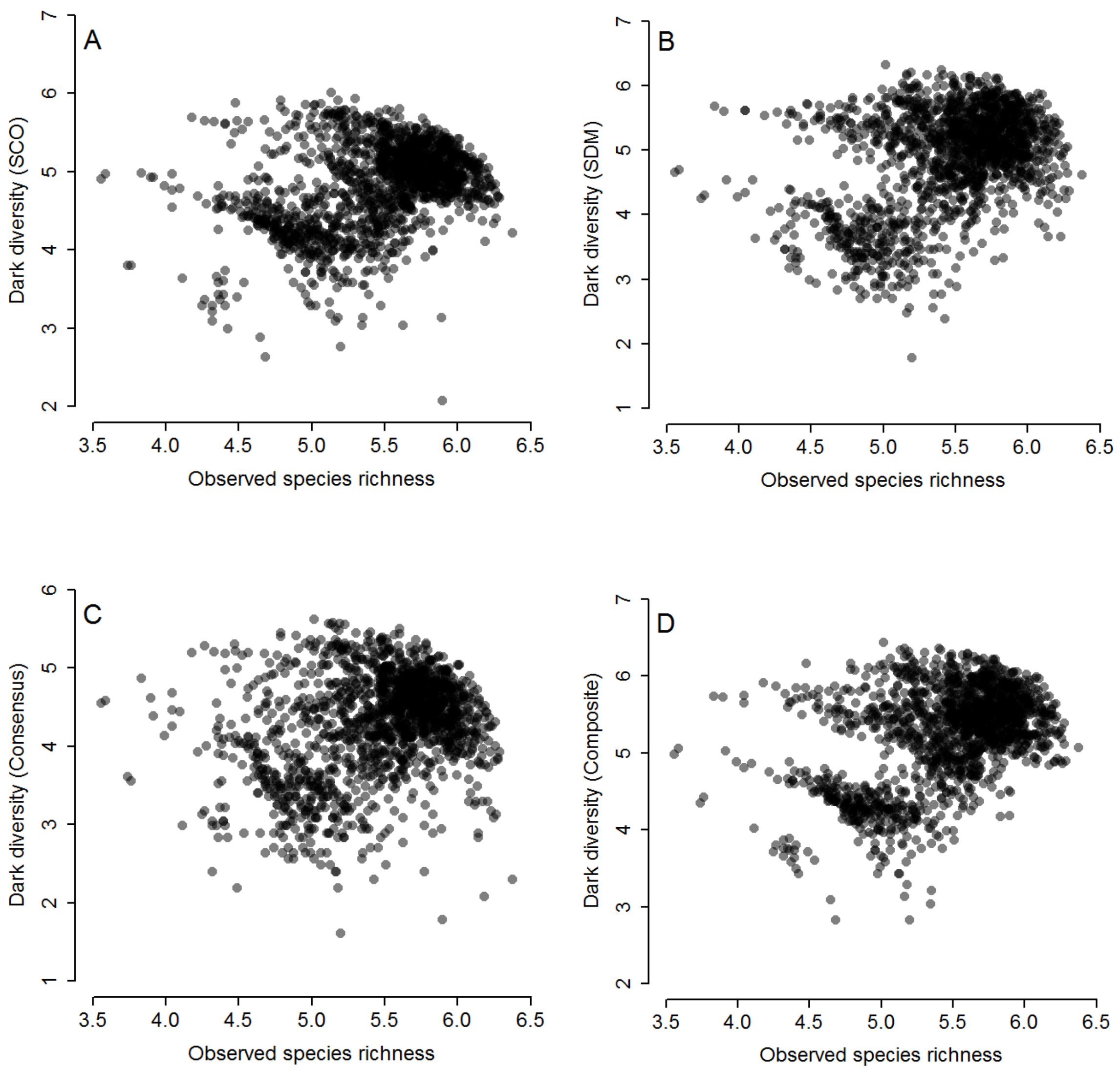

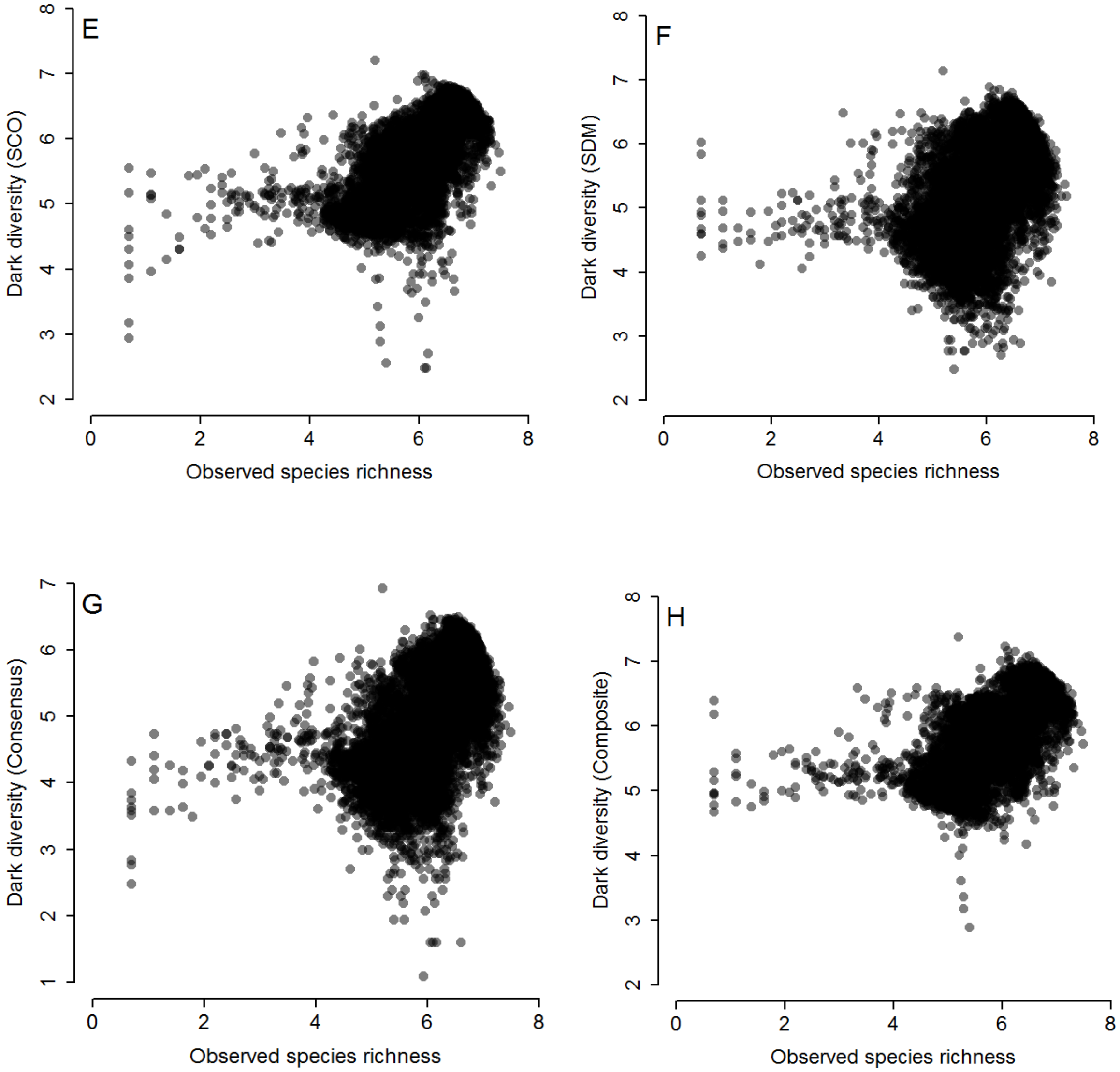

Figure S1 Scatterplots of observed species richness and: A) dark diversity estimated only by species co-occurrence (SCO) method ($t = 15.0$, $df = 1590$, $p < 0.001$, $r = 0.35$), B) dark diversity estimated only by species distribution modelling (SDM) method ($t = 17.1$, $df = 1590$, $p < 0.001$, $r = 0.39$), C) dark diversity (common species) estimated by both methods ($t = 11.0$, $df = 1590$, $p < 0.001$, $r = 0.27$), D) dark diversity (all species) estimated by both methods ($t = 18.6$, $df = 1590$, $p < 0.001$, $r = 0.42$), at the European scale (A,B,C,D) and E) dark diversity estimated only by species co-occurrence (SCO) method ($t = 94.0$, $df = 9313$, $p < 0.001$, $r = 0.7$), F) dark diversity

estimated only by species distribution modelling (SDM) method ($t = 55.0$, $df = 9313$, $p < 0.001$, $r = 0.5$), G) dark diversity (common species) estimated by both methods ($t = 65.3$, $df = 9313$, $p < 0.001$, $r = 0.56$), H) dark diversity (all species) estimated by both methods ($t = 87.8$, $df = 9313$, $p < 0.001$, $r = 0.67$) at the regional scale (E,F,G,H).

Appendix S3. Correlation between observed species richness and different completeness of site diversities.

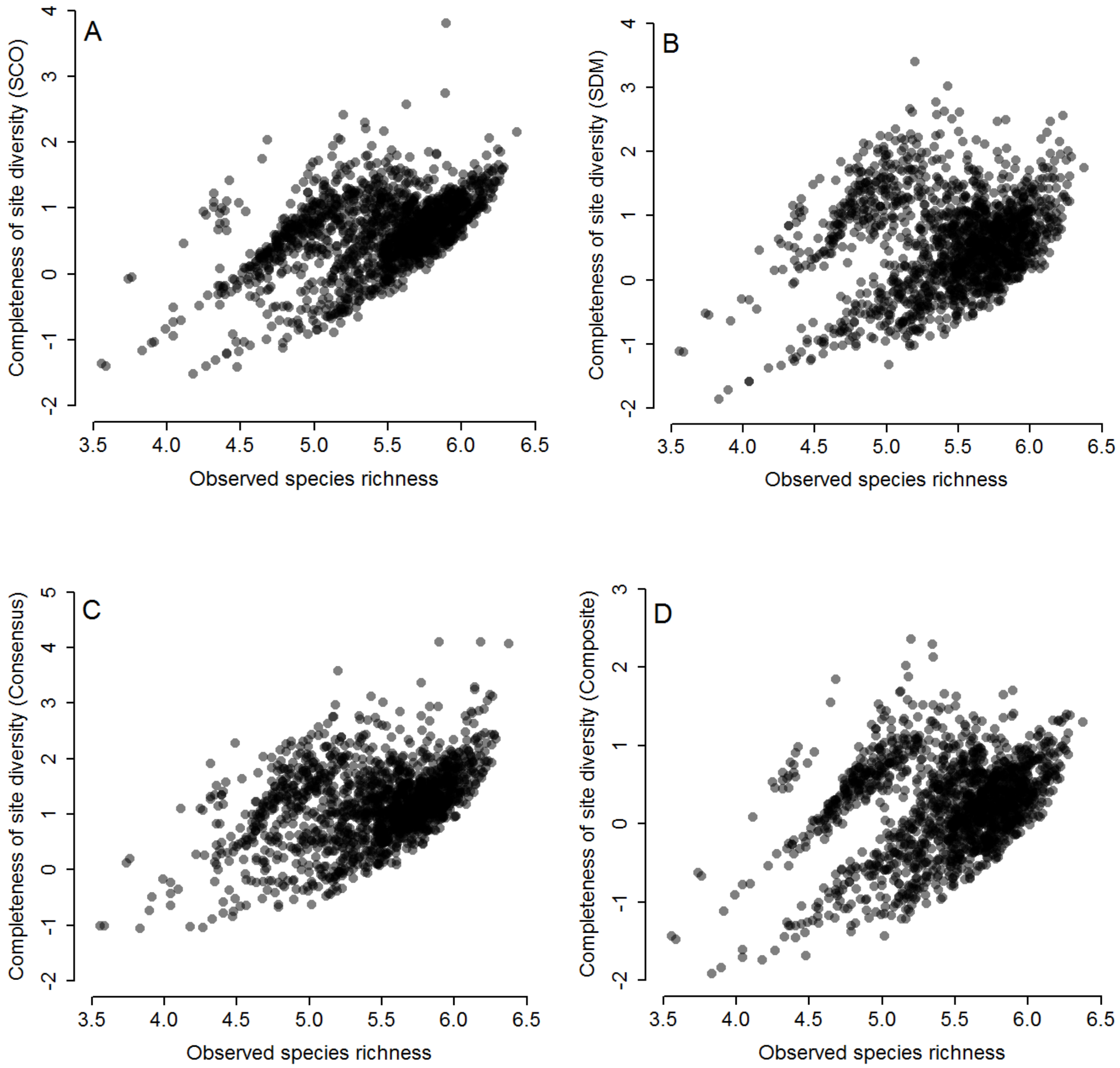

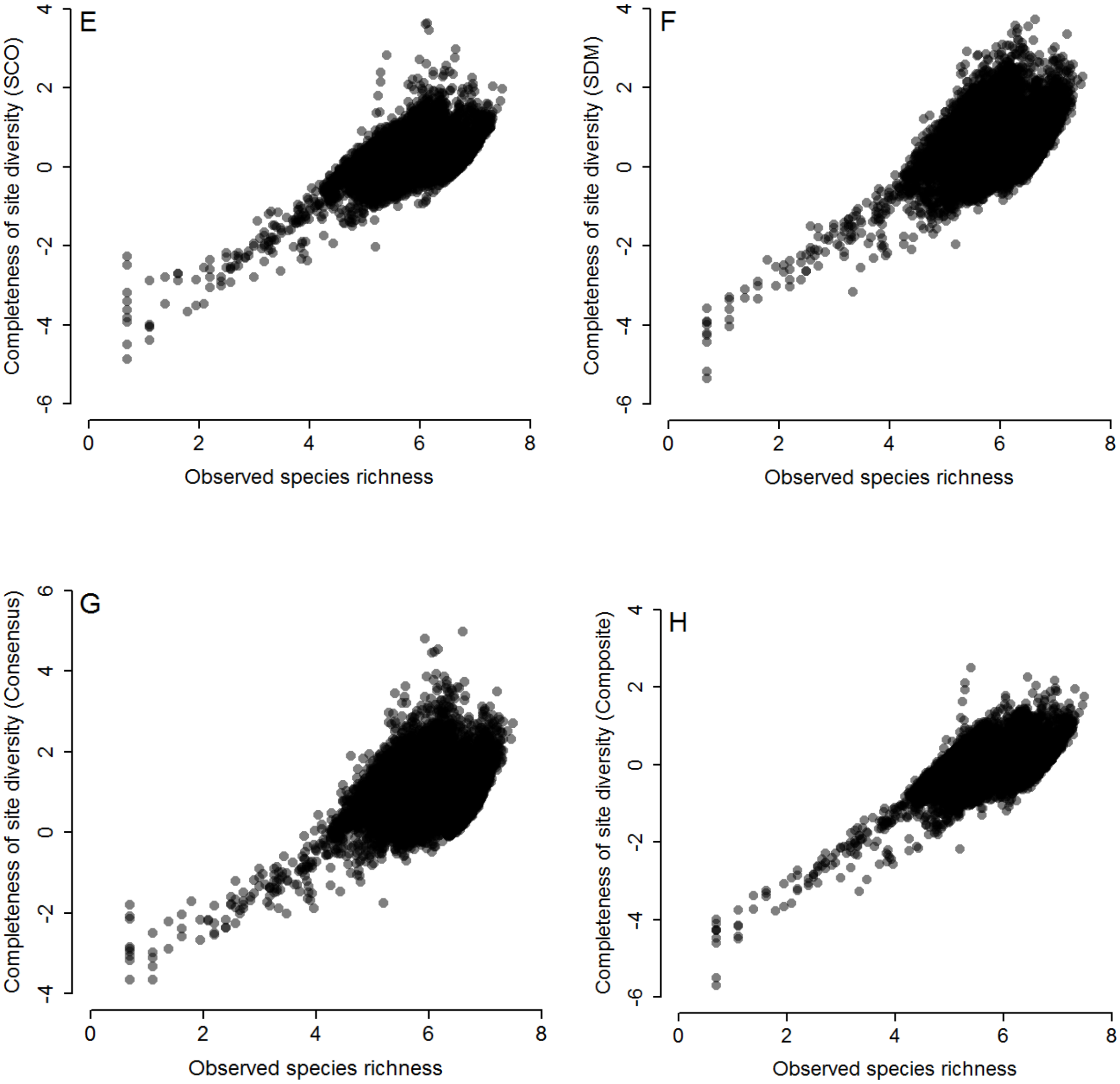

Figure S2 Scatterplots of observed species richness and: A) completeness of site diversity estimated only by species co-occurrence (SCO) method (t = 21.2, df = 1590, $p < 0.001$, r = 0.47), B) completeness of site diversity estimated only by species distribution modelling (SDM) method (t = 8.4, df = 1590, $p < 0.001$, r = 0.21), C) completeness of site diversity (common species) estimated by both methods (t = 18.3, df = 1590, $p < 0.001$, r = 0.42), D) completeness of site diversity (all species) estimated by both methods (t = 13.7, df = 1590, $p < 0.001$, r = 0.32),

at the European scale (A,B,C,D) and E) completeness of site diversity estimated only by species co-occurrence (SCO) method ($t = 61.9$, $df = 9313$, $p < 0.001$, $r = 0.54$), F) completeness of site diversity estimated only by species distribution modelling (SDM) method ($t = 50.3$, $df = 9313$, $p < 0.001$, $r = 0.46$), G) completeness of site diversity (common species) estimated by both methods ($t = 39.8$, $df = 9313$, $p < 0.001$, $r = 0.38$), H) completeness of site diversity (all species) estimated by both methods ($t = 74.4$, $df = 9313$, $p < 0.001$, $r = 0.61$) at the regional scale (E,F,G,H).

Appendix S4. Source and characteristics of the explanatory variables considered in this study.

Table S2 List of explanatory variables used in linear mixed effect models

Explanatory variable	Source	Original resolution	Observations
Climate			
**Altitude**	http://www.worldclim.org/current (Hijmans et al. 2005)	30 arc-seconds (~1 km)	Calculated mean and range values for each grid cell
**Mean Annual Temperature**	http://www.worldclim.org/bioclim (Hijmans et al. 2005)	30 arc-seconds (~1 km)	Calculated mean and range values for each grid cell
**Mean Annual Precipitation**	http://www.worldclim.org/bioclim (Hijmans et al. 2005)	30 arc-seconds (~1 km)	Calculated mean and range values for each grid cell
**Latitude**			
Geological properties			
**Geological Types**	http://geotypes.net/downloads.html (Durr et al. 2005)	0.5° x 0.5°	Count of different geological types within each grid cell
**Geological Heterogeneity**	http://geotypes.net/downloads.html (Durr et al. 2005)	0.5° x 0.5°	Heterogeneity was quantified by the Shannon index of different geological types for each grid cell
Soil properties			
**Soil pH**	http://soilgrids1km.isric.org/index.html (Hengl et al. 2014)	1 km	Calculated mean and range values for each grid cell
**Soil Organic Matter**	http://soilgrids1km.isric.org/index.html (Hengl et al. 2014)	1 km	Calculated mean and range values for each grid cell
**Soil Moisture**	http://nelson.wisc.edu/sage/data-and-models/atlas/maps.php (Willmott & Matsuura 2001)	0.5° x 0.5°	Calculated mean and range values for each grid cell
**Open Water**	http://www.eea.europa.eu/data-and-maps/data/corine-land-cover-2006-raster (Bossard et al. 2000)	250 m x 250 m	Calculated as log-ratio of Corine 2006 landuse types ln (open water/non-open water) for each grid cell
**Soil Heterogeneity**	http://soilgrids1km.isric.org/index.html (Hengl et al. 2014)	1 km	Heterogeneity was quantified by the Shannon index of different soil types for each grid cell
Plant growth conditions			
**Net Primary Productivity**	http://www.ntsg.umt.edu/project/mod17#data-product (Zhao et al. 2005)	30 arc-seconds (~1 km)	Calculated mean and range values for each grid cell
**Temperature Seasonality**	http://www.worldclim.org/bioclim (Hijmans et al. 2005)	30 arc-seconds (~1 km)	Calculated mean and range values for each grid cell
**Precipitation Seasonality**	http://www.worldclim.org/bioclim (Hijmans et al. 2005)	30 arc-seconds (~1 km)	Calculated mean and range values for each grid cell
Land use practices			
**Historical Cropland Area**	http://themasites.pbl.nl/tridion/en/themasites/hyde/ (Goldewijk et al. 2011)	5-arc-minute	Calculated as log-ratio of ln (Cropland area/non-cropland area) for year 1900 AD for each grid cell
**Current Cropland Area**	http://themasites.pbl.nl/tridion/en/themasites/hyde/ (Goldewijk et al. 2011)	5-arc-minute	Calculated as log-ratio of ln (Cropland area/non-cropland area) for year 2005 AD for each grid cell

**Cropland Area Change**	http://themasites.pbl.nl/tridion/en/themasites/hyde/ (Goldewijk et al. 2011)	5-arc-minute	Calculated as ln (Cropland area/non-cropland area) for year 1900 - ln (Cropland area/non-cropland area) for year 2005
**Historical Pasture Area**	http://themasites.pbl.nl/tridion/en/themasites/hyde/ (Goldewijk et al. 2011)	5-arc-minute	Calculated as log-ratio of ln (Pasture area/non-pasture area) for year 1900 AD for each grid cell
**Current Pasture Area**	http://themasites.pbl.nl/tridion/en/themasites/hyde/ (Goldewijk et al. 2011)	5-arc-minute	Calculated as log-ratio of ln (Pasture area/non-pasture area) for year 2005 AD for each grid cell
**Pasture Area Change**	http://themasites.pbl.nl/tridion/en/themasites/hyde/ (Goldewijk et al. 2011)	5-arc-minute	Calculated as ln (Pasture area/non-Pasture area) for year 1900 - ln (Pasture area/non-Pasture area) for year 2005
Ecoregions			
**Ecoregion Types**	http://www.worldwildlife.org/pages/conservation-science-data-and-tools (Olson et al. 2001)		Count of different Ecoregion types within each grid cell
**Distance to Closest Ecoregion**	http://www.worldwildlife.org/pages/conservation-science-data-and-tools (Olson et al. 2001)		Calculated from centroids of each grid cell to nearest ecoregion border

Bossard, M., Feranec, J. & Otahel, J. (2000) CORINE land cover technical guide: Addendum 2000. European Environment Agency Copenhagen.

Durr, H.H., Meybeck, M. & Durr, S.H. (2005) Lithologic composition of the Earth's continental surfaces derived from a new digital map emphasizing riverine material transfer. *Global Biogeochemical Cycles,* **19**.

Goldewijk, K.K., Beusen, A., van Drecht, G. & de Vos, M. (2011) The HYDE 3.1 spatially explicit database of human-induced global land-use change over the past 12,000 years. *Global Ecology and Biogeography,* **20,** 73-86.

Hengl, T., de Jesus, J.M., MacMillan, R.A., Batjes, N.H., Heuvelink, G.B.M., Ribeiro, E., Samuel-Rosa, A., Kempen, B., Leenaars, J.G.B., Walsh, M.G. & Gonzalez, M.R. (2014) SoilGrids1km-Global Soil Information Based on Automated Mapping. *Plos One,* **9**.

Hijmans, R.J., Cameron, S.E., Parra, J.L., Jones, P.G. & Jarvis, A. (2005) Very high resolution interpolated climate surfaces for global land areas. *International Journal of Climatology,* **25,** 1965-1978.

Olson, D.M., Dinerstein, E., Wikramanayake, E.D., Burgess, N.D., Powell, G.V., Underwood, E.C., D'amico, J.A., Itoua, I., Strand, H.E. & Morrison, J.C. (2001) Terrestrial Ecoregions of the World: A New Map of Life on Earth A new global map of terrestrial ecoregions provides an innovative tool for conserving biodiversity. *BioScience*, **51,** 933-938.

Willmott, C.J. & Matsuura, K. (2001) Terrestrial water budget data archive: monthly time series (1950–1999). *Center for Climatic Research, University of Delaware, Newark, DE*.

Zhao, M.S., Heinsch, F.A., Nemani, R.R. & Running, S.W. (2005) Improvements of the MODIS terrestrial gross and net primary production global data set. *Remote Sensing of Environment*, **95,** 164-176.

Appendix S5. Correlation matrices from PCA analyses.

Table S3 Relationships between explanatory variables and first three principal components at the European scale.

	PCA 1 (Heterogeneity)	PCA 2 (Latitude)	PCA 3 (Seasonality)
Mean Altitude	0.78	-0.29	0.25
Altitudinal Range	0.88	-0.32	0.17
Mean Annual Temperature	-0.18	-0.79	-0.25
Annual Temperature Range	0.88	-0.31	0.18
Mean Annual Precipitation	0.68	0.36	-0.37
Annual Precipitation range	0.86	-0.16	-0.09
Latitude	-0.30	0.88	0.02
Geological Types	0.55	-0.18	0.12
Geological Heterogeneity	0.52	-0.19	0.12
Mean Soil pH	-0.30	-0.87	0.09
Soil pH Range	0.14	-0.31	0.33
Mean Soil Organic Matter	0.36	0.88	-0.06
Soil Organic Matter Range	0.18	0.81	0.00
Mean Soil Moister	0.65	0.49	-0.31
Soil Moister Range	0.10	0.04	0.00
Open Water	-0.31	0.45	0.20
Soil Heterogeneity	-0.45	-0.06	0.00
Mean Net Primary Productivity	0.38	0.24	-0.54
Net Primary Productivity Range	0.52	-0.01	-0.41
Mean Temperature Seasonality	-0.38	0.15	0.70
Temperature Seasonality Range	0.68	-0.10	0.31
Mean Precipitation Seasonality	-0.06	-0.26	0.61
Precipitation Seasonality Range	0.64	-0.22	0.13
Historical Cropland Area	-0.68	-0.30	-0.23
Current Cropland Area	-0.53	-0.59	-0.21
Cropland Area Change	0.50	-0.04	0.15
Historical Pasture Area	0.09	-0.50	-0.60
Current Pasture Area	-0.06	-0.56	-0.46
Pasture Area Change	-0.25	0.05	0.38
Ecoregion Types	0.36	-0.28	0.18
Distance to Closest Ecoregion	-0.37	0.29	-0.17

Table S4 Relationships between explanatory variables and first two principal components at the regional scale.

	PCA 1 (Latitude)	PCA 2 (Heterogeneity)
Mean Altitude	-0.39	0.72
Altitudinal Range	-0.68	0.65
Mean Annual Temperature	-0.74	-0.60
Annual Temperature Range	-0.69	0.65
Mean Annual Precipitation	-0.61	0.30
Annual Precipitation range	-0.83	0.42
Latitude	0.91	0.26
Geological Types	-0.14	0.24
Geological Heterogeneity	-0.12	0.21
Mean Soil pH	-0.63	-0.5
Soil pH Range	-0.14	0.08
Mean Soil Organic Matter	0.61	0.55
Soil Organic Matter Range	0.56	0.46
Soil Heterogeneity	-0.13	-0.16
Mean Net Primary Productivity	-0.62	-0.41
Net Primary Productivity Range	-0.53	0.09
Mean Temperature Seasonality	0.80	0.28
Temperature Seasonality Range	-0.47	0.61
Mean Precipitation Seasonality	0.55	0.33
Precipitation Seasonality Range	-0.56	0.48
Ecoregion Types	-0.26	-0.06
Distance to Closest Ecoregion	0.60	0.15

Appendix S6. Spatially informed linear mixed effect model results — Observed species richness and different dark diversity and completeness of site diversity estimates (European and regional scale) related to natural and anthropogenic factors.

Table S5. Summary results of the spatially-informed linear mixed effect model linking observed species richness to natural and anthropogenic factors at the European scale (d.f.= 1213).

	Slope	SE	t-value	p-value
Heterogeneity (PCA 1)	0.139	0.014	10.3	<0.0001
Latitude (PCA 2)	0.033	0.022	1.5	0.1251
Seasonality (PCA 3)	0.084	0.029	2.9	0.0039

Table S6. Summary results of the spatially-informed linear mixed effect model linking observed species richness to natural and anthropogenic factors at the regional scale (d.f.= 10274).

	Slope	SE	t-value	p-value
Latitude (PCA 1)	0.120	0.015	8.1	<0.0001
Heterogeneity (PCA 2)	-0.035	0.011	-3.2	0.0015

Table S7. Summary results of the spatially-informed linear mixed effect model linking dark diversity (SCO) to natural and anthropogenic factors at the European scale (d.f.= 1213).

	Slope	SE	t-value	p-value
Heterogeneity (PCA 1)	-0.001	0.014	-0.1	0.9237
Latitude (PCA 2)	-0.019	0.023	-0.8	0.3959

Seasonality (PCA 3)	-0.145	0.030	-4.8	<0.0001

Table S8. Summary results of the spatially-informed linear mixed effect model linking dark diversity (SCO) to natural and anthropogenic factors at the regional scale (d.f.= 10274).

	Slope	SE	t-value	p-value
Latitude (PCA 1)	0.118	0.011	11.2	<0.0001
Heterogeneity (PCA 2)	-0.150	0.008	-19.6	<0.0001

Table S9. Summary results of the spatially-informed linear mixed effect model linking dark diversity (SDM) to natural and anthropogenic factors at the European scale (d.f.= 1213).

	Slope	SE	t-value	p-value
Heterogeneity (PCA 1)	0.022	0.014	1.5	0.1242
Latitude (PCA 2)	-0.1	0.024	-4.1	<0.0001
Seasonality (PCA 3)	-0.097	0.032	-3.1	0.0023

Table S10. Summary results of the spatially-informed linear mixed effect model linking dark diversity (SDM) to natural and anthropogenic factors at the regional scale (d.f.= 10274).

	Slope	SE	t-value	p-value
Latitude (PCA 1)	-0.004	0.011	-0.4	0.7051
Heterogeneity (PCA 2)	-0.1	0.008	-12.5	<0.0001

Table S11. Summary results of the spatially-informed linear mixed effect model linking consensus dark diversity to natural and anthropogenic factors at the European scale (d.f.= 1213).

	Slope	SE	t-value	p-value
Heterogeneity (PCA 1)	-0.009	0.016	-0.6	0.5747
Latitude (PCA 2)	0.05	0.026	1.9	0.0553
Seasonality (PCA 3)	-0.165	0.035	-4.7	<0.0001

Table S12. Summary results of the spatially-informed linear mixed effect model linking consensus dark diversity to natural and anthropogenic factors at the regional scale (d.f.= 10274).

	Slope	SE	t-value	p-value
Latitude (PCA 1)	0.038	0.011	3.6	0.0003
Heterogeneity (PCA 2)	-0.119	0.008	-15.4	<0.0001

Table S13. Summary results of the spatially-informed linear mixed effect model linking composite dark diversity to natural and anthropogenic factors at the European scale (d.f.= 1213).

	Slope	SE	t-value	p-value
Heterogeneity (PCA 1)	0.024	0.012	2.0	0.0507
Latitude (PCA 2)	-0.114	0.021	-5.3	<0.0001
Seasonality (PCA 3)	-0.112	0.028	-4.0	0.0001

Table S14. Summary results of the spatially-informed linear mixed effect model linking composite dark diversity to natural and anthropogenic factors at the regional scale (d.f.= 10274).

	Slope	SE	t-value	p-value
Latitude (PCA 1)	0.072	0.009	8.4	<0.0001
Heterogeneity (PCA 2)	-0.133	0.006	-21.1	<0.0001

Table S15. Summary results of the spatially-informed linear mixed effect model linking completeness of site diversity (SCO) to natural and anthropogenic factors at the European scale (d.f.= 1213).

	Slope	SE	t-value	p-value
Heterogeneity (PCA 1)	0.082	0.017	4.9	<0.0001
Latitude (PCA 2)	0.004	0.025	0.2	0.8789
Seasonality (PCA 3)	0.151	0.034	4.4	<0.0001

Table S16. Summary results of the spatially-informed linear mixed effect model linking completeness of site diversity (SCO) to natural and anthropogenic factors at the regional scale (d.f.= 10274).

	Slope	SE	t-value	p-value
Latitude (PCA 1)	0.038	0.021	1.8	0.0684
Heterogeneity (PCA 2)	0.1	0.015	6.5	<0.0001

Table S17. Summary results of the spatially-informed linear mixed effect model linking completeness of site diversity (SDM) to natural and anthropogenic factors at the European scale (d.f.= 1213).

	Slope	SE	t-value	p-value
Heterogeneity (PCA 1)	0.048	0.016	3.0	0.0024
Latitude (PCA 2)	0.054	0.026	2.0	0.0407
Seasonality (PCA 3)	0.127	0.035	3.6	0.0003

Table S18. Summary results of the spatially-informed linear mixed effect model linking completeness of site diversity (SDM) to natural and anthropogenic factors at the regional scale (d.f.= 10274).

	Slope	SE	t-value	p-value
Latitude (PCA 1)	0.11	0.021	5.3	<0.0001
Heterogeneity (PCA 2)	0.056	0.015	3.8	0.0002

Table S19. Summary results of the spatially-informed linear mixed effect model linking consensus completeness of site diversity to natural and anthropogenic factors at the European scale (d.f.= 1213).

	Slope	SE	t-value	p-value
Heterogeneity (PCA 1)	0.074	0.016	4.5	<0.0001
Latitude (PCA 2)	-0.05	0.023	-2.2	0.0298
Seasonality (PCA 3)	0.130	0.032	4.0	0.0001

Table S20. Summary results of the spatially-informed linear mixed effect model linking consensus completeness of site diversity to natural and anthropogenic factors at the regional scale (d.f.= 10274).

	Slope	SE	t-value	p-value
Latitude (PCA 1)	0.075	0.020	3.7	0.0002
Heterogeneity (PCA 2)	0.082	0.015	5.7	0.0001

Table S21. Summary results of the spatially-informed linear mixed effect model linking composite completeness of site diversity to natural and anthropogenic factors at the European scale (d.f.= 1213).

	Slope	SE	t-value	p-value
Heterogeneity (PCA 1)	0.06	0.016	3.8	0.0001
Latitude (PCA 2)	0.062	0.026	2.4	0.0164
Seasonality (PCA 3)	0.141	0.034	4.1	<0.0001

Table S22. Summary results of the spatially-informed linear mixed effect model linking composite completeness of site diversity to natural and anthropogenic factors at the regional scale (d.f.= 10274).

	Slope	SE	t-value	p-value
Latitude (PCA 1)	0.08	0.022	3.7	0.0002
Heterogeneity (PCA 2)	0.075	0.016	4.7	<0.0001
